# A Multi-Task Representation Learning Architecture for Enhanced Graph Classification

**DOI:** 10.3389/fnins.2019.01395

**Published:** 2020-01-09

**Authors:** Yu Xie, Maoguo Gong, Yuan Gao, A. K. Qin, Xiaolong Fan

**Affiliations:** ^1^Key Laboratory of Intelligent Perception and Image Understanding of Ministry of Education, School of Electronic Engineering, Xidian University, Xi'an, China; ^2^Department of Computer Science and Software Engineering, Swinburne University of Technology, Melbourne, VIC, Australia

**Keywords:** multi-task learning, representation learning, graph classification, node classification, graph neural network

## Abstract

Composed of nodes and edges, graph structured data are organized in the non-Euclidean geometric space and ubiquitous especially in chemical compounds, proteins, etc. They usually contain rich structure information, and how to effectively extract inherent features of them is of great significance on the determination of function or traits in medicine and biology. Recently, there is a growing interest in learning graph-level representations for graph classification. Existing graph classification strategies based on graph neural networks broadly follow a single-task learning framework and manage to learn graph-level representations through aggregating node-level representations. However, they lack the efficient utilization of labels of nodes in a graph. In this paper, we propose a novel multi-task representation learning architecture coupled with the task of supervised node classification for enhanced graph classification. Specifically, the node classification task enforces node-level representations to take full advantage of node labels available in the graph and the graph classification task allows for learning graph-level representations in an end-to-end manner. Experimental results on multiple benchmark datasets demonstrate that the proposed architecture performs significantly better than various single-task graph neural network methods for graph classification.

## 1. Introduction

Learning with graph-structured data, such as chemical compounds or proteins, requires effective representations of their internal structure (Hamilton et al., 2017b), as the structural changes usually have an impact on the traits they express. Nodes with different properties and unique connections make up a variety of graphs, and one of the graph learning tasks is to predict the labels for graphs. Specifically, nodes represent entities and edges represent relationships between them, and the category of a graph is always correlated with the graph structure and node labels in real world. Therefore, models capable of capturing node features and graph structure have been shown to achieve superior performances on classification tasks (Rossi et al., 2012).

In recent years, there has been a surge of interest in Graph Neural Networks (GNNs) (Cao et al., 2016; Monti et al., 2017; Schlichtkrull et al., 2018; Zou and Lerman, 2019) for learning representations of graphs and nodes. The general approach with GNNs broadly follows a recursive neighborhood aggregation scheme by passing, transforming and aggregating feature vectors of nodes across the graph (Gilmer et al., 2017; Hamilton et al., 2017a; Xu et al., 2018). Empirically, these GNNs have achieved outstanding performance in many tasks such as graph classification and node classification. However, a major limitation of these GNN architectures is that they only focus on a specific task and their design is based on heuristics or experimental trial-and-error, and there is little theoretical understanding of the properties. As a result, GNNs' representational capacity and generalization ability are limited (Xu et al., 2019).

In real-world applications, the graph classification task is always correlated with the node classification task, and effective node representations are conducive to learning graph features with the same aggregation scheme (Petar et al., 2018). For example, a graph classification task is to predict the carcinogenicity of proteins, for which categories of nodes that represent different amino acids are of crucial importance. Nevertheless, previous related deep graph embedding methods treat real problems as several single tasks, while ignoring the rich correlation information between these related tasks. They do not follow human's cognitive laws of new things that people often apply the knowledge they have acquired by learning related tasks, whereas working on a single task from scratch is inefficient and increases the risk of overfitting. Moreover, they usually require multiple training steps that are difficult to optimize for each task (Tran, 2018).

**Figure 1:**
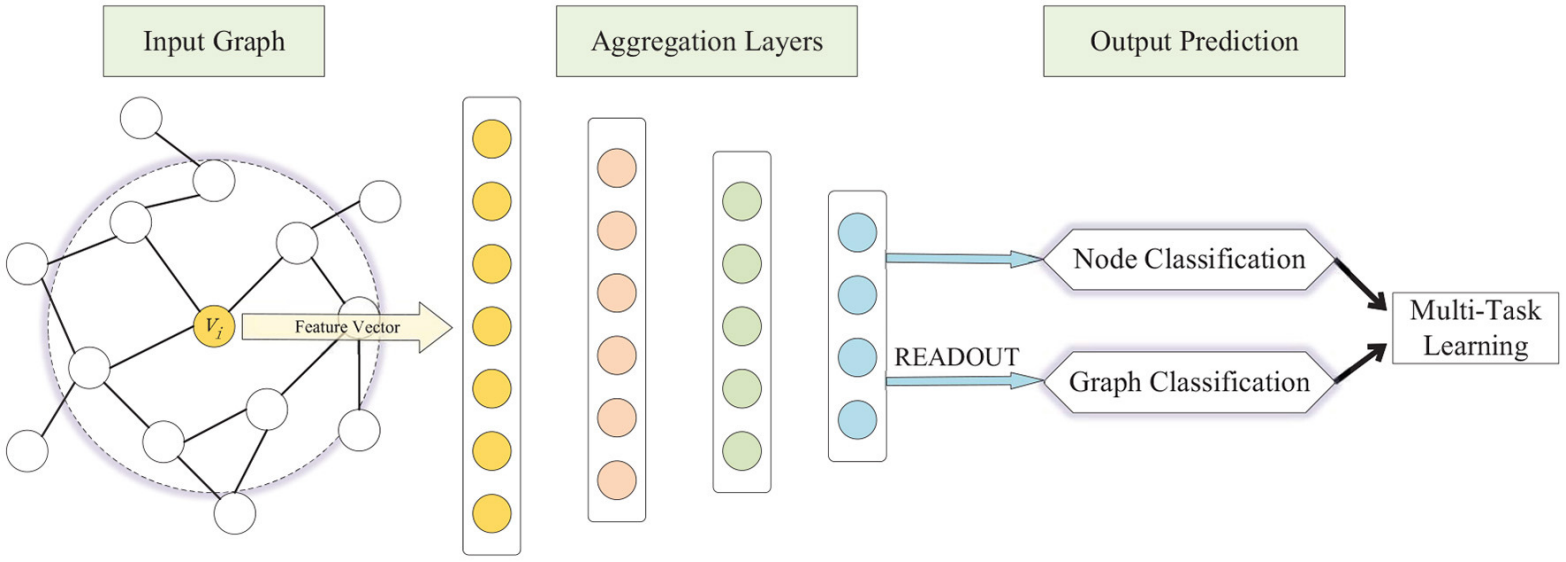
Schematic depiction of the Multi-Task Representation Learning (MTRL) architecture.

To address the aforementioned challenges, we present a multi-task representation learning (MTRL) framework for both graph classification and node classification, schematically depicted in Figure 1. The MTRL framework is capable of learning representations of latent node embeddings and graph embeddings from local graph topology, and the shared representations between different tasks enable our model to generalize better on each task. A densely connected neural network is trained end-to-end to learn embeddings for nodes and graphs from the adjacency vector or feature vector, in which the READOUT function aggregates node representations from the final iteration to generate the entire graph's representation. The weighted sum of losses of graph classification and node classification is utilized in the back propagation of the multi-task learning process, thus graph-level features and fine-grained node features can be captured synchronously, and the generalization ability of models is improved through collaborative training. Specifically, our contributions in this paper are as follows:

- We propose a novel multi-task representation learning architecture and extend it further for different models designed specifically for graph classification. Compared with single-task learning models, our approach shows better performance in different tasks.
- Our architecture is efficiently trained end-to-end for the joint and simultaneous multi-task learning of supervised graph classification and node classification in a single stage.
- We conduct empirical evaluation of our architecture on five challenging benchmark graph-structured datasets, and the experimental results demonstrate significant improvement over state-of-the-art baselines.

The full text is structured as follows. After a basic introduction, the related backgrounds and algorithms about GNNs are shown in section 2. In section 3, we give a clear definition of the graph classification and the node classification, then the MTRL architecture is developed. Section 4 provides the experimental results of two classification tasks. Finally, in section 6 we conclude with a discussion of our architecture and summarize the future work.

## 2. Related Work

Representation learning (Bengio et al., 2013) has been widely utilized in various fields such as computer vision (Du and Wang, 2015; Butepage et al., 2017) and natural language processing (Janner et al., 2018). With the rapid development of biology, chemistry, and medical science, the microscopic structure of molecular compounds as proteins and genes are paid more attention. This kind of graph-structured data attracts the interests of researchers in graph classification, and various methods are presented to learn graph representations.

Recently, a wide variety of GNN models have been proposed, including approaches inspired by convolutional neural networks (Defferrard et al., 2016; Kipf and Welling, 2016; Lei et al., 2017), recursive neural networks (Scarselli et al., 2008) and recurrent neural networks (Li et al., 2016). These methods have been applied to various tasks, such as graph classification (Dai et al., 2016; Zhang et al., 2018) and node classification (Kipf and Welling, 2016; Hamilton et al., 2017a). Instead of using hand-crafted features suited for specific tasks, deep learning techniques enable models to automatically learn features and representations for each node. In the context of graph classification, which is our main task, the major challenge is going from node embeddings to the representation of the entire graph. Most methods (Duvenaud et al., 2015; Li et al., 2016; Gilmer et al., 2017) have the limitation that they simply pool all the node embeddings in a single layer and do not learn the hierarchical representations, so they are unable to capture the natural structures of large graphs. Some recent approaches have focused on alleviating this problem by adopting novel aggregation approaches.

A latest research (Xu et al., 2019) developed theoretical foundations for reasoning about the expressive power of GNNs and presented a Graph Isomorphism Network (GIN) under the neighborhood aggregation framework. They proved that GNNs are at most as powerful as the Weisfeiler-Lehman (WL) test in distinguishing graph structures, and showed the discriminative power of GIN is equal to that of the WL test. They developed a “deep multisets" theory, which parameterizes universal multiset functions with the neural network, and a multiset is a generalized concept of a set that allows elements in it have multiple instances. Besides, multi-layer perceptrons (MLPs) are utilized in the model so that different graph structures could be discriminated through aggregation, combination and READOUT strategy. GIN updates node representations as:

(1) $$\begin{array}{l} h_{v}^{\left(l\right)}=MLP^{\left(l\right)}\left(\left(1+\epsilon^{\left(l\right)}\right)\cdot h_{v}^{\left(l-1\right)}+\sum_{u\in N\left(v\right)}h_{u}^{\left(l-1\right)}\right). \end{array}$$

They applied the sum aggregator that adds all neighbors of the current node, and set the combination method as (1 + ϵ^(*l*)^) in *l*th layer, so that all nodes can be effectively integrated and mapped to the next layer. As a theoretical framework, GIN outperforms popular GNN variants, while some other researchers focus on coarsening the input graph inspired by the pooling method in convolutional neural networks.

DIFFPOOL (Ying et al., 2018) is a differentiable graph pooling module that can be adapted to various GNN architectures in a hierarchical and end-to-end fashion. DIFFPOOL learns a cluster assignment for nodes at each layer, which then forms the coarsened input for the next layer, and it is able to extract the complex hierarchical structure of graphs. Given the input adjacency matrix and node embedding matrix, the DIFFPOOL layer coarsens the input graph and generates a coarsened adjacency matrix as well as a new embedding matrix for each node or clusters in the coarsened graph. In particular, they applied the two following equations:

(2) $$\begin{array}{l} X^{\left(l+1\right)}=S^{{\left(l\right)}^{T}}Z^{\left(l\right)}\in {\mathbb{R}}^{n_{l+1}\times d}, \end{array}$$

(3) $$\begin{array}{l} A^{\left(l+1\right)}=S^{{\left(l\right)}^{T}}A^{\left(l\right)}S^{\left(l\right)}\in {\mathbb{R}}^{n_{l+1}\times n_{l+1}}, \end{array}$$

where *A*^(*l*)^ represents the adjacency matrix at this layer. *Z*^(*l*)^ and *X*^(*l*)^ denote the input node embedding matrix and the cluster embedding matrix respectively. *S*^(*l*)^ is the probabilistic assignment matrix that assigns each node at layer *l* to a specific cluster in the next coarsened layer *l* + 1. Each row of *S*^(*l*)^ corresponds to a node or cluster at layer *l*, and each column corresponds to a target cluster at layer *l* + 1. The assignment matrix is generated from the pooling GNN using input cluster features *X*^(*l*)^ and the cluster adjacency matrix *A*^(*l*)^:

(4) $$\begin{array}{l} S^{\left(l\right)}=softmax\left(GNN_{l,pool}\left(A^{\left(l\right)},X^{\left(l\right)}\right)\right), \end{array}$$

where the softmax function is utilized in a row-wise fashion. The output dimension of *GNN*_*l,pool*_ is pre-defined as the hyperparameter of the model, which corresponds to the maximum number of clusters in each layer. Besides, the embedding GNN is a standard GNN module applied to *A*^(*l*)^ and *X*^(*l*)^:

(5) $$\begin{array}{l} Z^{\left(l\right)}=GNN_{l,embed}\left(A^{\left(l\right)},X^{\left(l\right)}\right). \end{array}$$

The adjacency matrix between the cluster nodes *A*^(*l*)^ from Equation (3) and the pooled features for clusters *X*^(*l*)^ from Equation (2) are passed through a standard GNN to obtain new embeddings *Z*^(*l*)^ for the cluster nodes. GIN and DIFFPOOL can learn to discriminate and capture the meaningful structure of graphs in terms of aggregation and pooling, respectively, and they are powerful in the graph classification task.

In many real-world applications, such as network analysis and molecule classification, the input data is observed with a fraction of labeled graphs and labeled nodes. Thus it is desirable for the model to predict the labels of graphs and nodes simultaneously in a multi-task learning setting. Multi-task learning (MTL) refers to the paradigm of learning several related tasks together, which has been broadly used in natural language processing (Chen et al., 2018; Schulz et al., 2018; Sanh et al., 2019), computer vision (Choi et al., 2018; Kendall et al., 2018; Liu et al., 2019) and genomics (Yang et al., 2018). To be specific, SaEF-AKT (Huang et al., 2019) introduces a general similarity measure and an adaptive knowledge transfer mechanism to assist the knowledge transfer among tasks. EMT (Evolutionary multitasking) via autoencoding (Feng et al., 2018) allows the incorporation of multiple search mechanisms with different biases in the EMT paradigm. MTL is inspired by human learning activities where people could transfer the knowledge learned from the previous problems to facilitate learning a new task. Similar to human learning, the knowledge contained in a problem can be leveraged by related problems in the multi-task machine learning process. A main assumption of MTL is that there is an optimal shared parameter space for all problems, which is regularized by a specific loss, manually defined relationships or other automatic methods that estimate the latent structure of relationships among problems. Due to the shared processes that give rise to strong dependencies of multiple tasks, the MTL approach is able to explore and leverage the commonalities among related tasks in the learning process.

## 3. Methodology

The key idea of the MTRL architecture is that it enables the graph classification and node classification tasks to be performed simultaneously. Along the way, it helps to improve the generalization ability of the model and avoid falling into the local minimum. In this section, we outline the MTRL structure and demonstrate how it works on the GIN and DIFFPOOL models. Before introducing the architecture, we start by discussing the statement of the problem.

### 3.1. Problem Statement

The input to the MTRL architecture is a set of labeled graphs $D=\left\{\left(G_{1},y_{1}\right),\left(G_{2},y_{2}\right),\ldots \right\}$, where $y_{i}\in Y$ is the label associated with graph $G_{i}\in G$, and *G* = (*A, F, V*) denotes a graph with an adjacency matrix *A* ∈ {0, 1}^*n*×*n*^ and node feature vectors *F* ∈ ℝ^*n*×*d*^, assuming each node *v* ∈ *V* has *d* features. There are two tasks of interest: (1) *Graph classification*, where graph labels *y*_*G*_ are given and the goal is to learn a representation vector *r*_*G*_ that helps predict the label of the graph, *y*_*G*_ = *g*(*r*_*G*_); (2) *Node classification*, where each node *v* has a corresponding label *y*_*v*_ and we aim to learn a representation vector *r*_*v*_ such that *v*′*s* label could be predicted as *y*_*v*_ = *h*(*r*_*v*_). The main symbols are listed in Table 1.

**Table 1:** Main symbols and descriptions in the paper.

**Notations**	**Descriptions**
*G*	Input labeled graph
*A*	Adjacency matrix
*F*	Feature information matrix
*n*	Number of nodes in a graph
*d*	Dimension of node features
*r*_*G*_	Graph embedding representation
*r*_*v*_	Node embedding representation

### 3.2. Multi-Task Representation Learning

In this work, we build upon the MTRL architecture to learn useful representations for graph classification and node classification in an end-to-end fashion. The graph classification is set as the primary task while the node classification as the secondary task, and the performance of the model could be improved by sharing the training information in the primary task and the auxiliary related task. Since these two classification tasks are related, it is intuitive to assume that they share a common feature representation based on the original features, which do not have enough expressive power for multiple tasks. A more powerful representations could be learned for both tasks by the MTRL architecture and it will bring improvement on the performance.

Follow the GNN structure, the architecture adopts a neighborhood aggregation and combination strategy, where the representation of a node is iteratively updated by aggregating its neighbors' representations and combining its representation of the previous layer. Especially, after *k* iterations of aggregation and combination, representations of each node is able to capture the structural information within its *k*-hop graph neighborhood. For node classification, the node representation of the final layer is utilized for prediction. For graph classification, there should be a READOUT method that aggregates all node representations of the final iteration to generate the graph representation.

Based on the normal GNN models for graph classification, the MTRL architecture adds an additional softmax layer for node classification. Given an input graph *G*, the parameters of the model are trained to minimize the cross-entropy of the predicted and true distributions,

(6) $$\begin{array}{l} L_{v}=-\sum_{v\in V}\sum_{c\in C}y_{v}^{c}\cdot log\left({ŷ}_{v}^{c}\right) \end{array}$$

where $y_{v}^{c}$ is the ground-truth label; ${ŷ}_{v}^{c}$ is prediction probabilities, and C indicates node classes. The loss of graph classification $L_{G}$ is similar to Equation (6).

During the multi-task learning process, the related information is exchanged and supplemented by a shared representation at a shallow level, and the accuracy of node classification and graph classification are optimized simultaneously. The node classification task enforces node-level representations to take full advantage of node labels available in the graph and the graph classification task allows for learning graph-level representations in an end-to-end manner. More precisely, we achieve multi-task learning on graphs by designing a joint loss function that combines the two masked categorical cross-entropy losses for supervised graph classification and node classification:

(7) $$\begin{array}{l} L_{MTRL}=L_{G}+\alpha \cdot L_{v} \end{array}$$

where α is used for the integration of the loss so that the scale of all losses is close. Noted that when α is 0, the architecture is equal to a single-task graph classification model. Besides, how we extract node representations is crucial to the discrimination task. In particular, we consider two state-of-the-art models that employ the above MTRL architecture.

#### 3.2.1. Multi-Task GIN

The original GIN applies five GNN layers and all MLPs have two layers. It utilizes information from all depths of the model to consider all structural information in Equation (8), because features from deep layers are key to achieving better discriminative performance while features from shallow layers could generalize better.

(8) $$\begin{array}{l} r_{G}=CONCAT\left(READOUT\left(\left\{r_{v}^{\left(l\right)}|v\in G\right\}\right)|l=0,1,\ldots ,L\right). \end{array}$$

The READOUT is set as a simple permutation invariant function such as summation. Similarly, to obtain both global and refined representations of nodes, we achieve node features extraction that concatenated across all layers as follows, and then the softmax activation function is used to produce a probability distribution over node labels.

(9) $$\begin{array}{l} r_{v}=CONCAT\left(r_{v}^{\left(l\right)}|l=0,1,\ldots ,L\right). \end{array}$$

In the multi-task GIN (MT-GIN), all parameters in the network except for two softmax layers are shared. Considering that different tasks may have various sample noises in all directions with different patterns, the hard parameter sharing method could offset some noises through learning from multiple tasks, which will result in better performance on each task.

#### 3.2.2. Multi-Task DIFFPOOL

**Figure 2:**
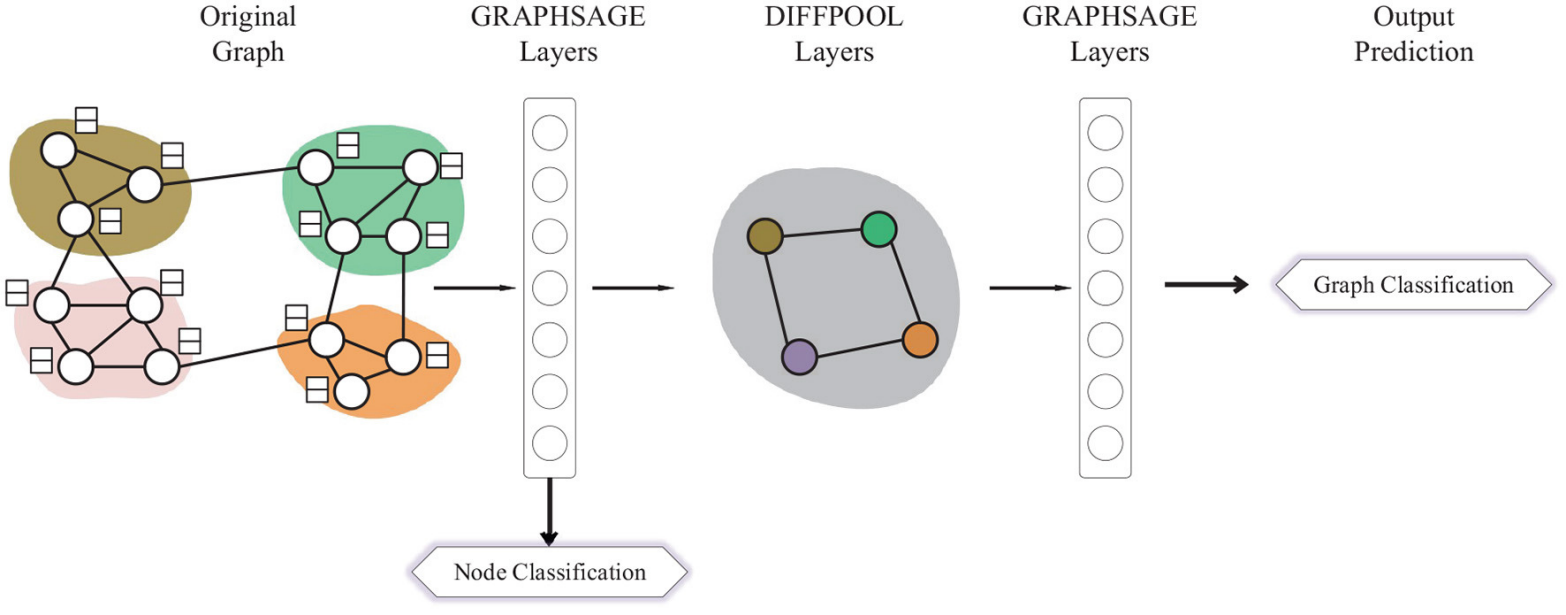
A graphical illustration of the Multi-task DIFFPOOL model.

Different from GIN, DIFFPOOL applies a more sophisticated graph-level pooling READOUT function. The GNN model used for DIFFPOOL is built on top of the GRAPHSAGE (Hamilton et al., 2017a) architecture as it has superior performance compared with the standard graph convolutional network. It sets a DIFFPOOL layer after two GRAPHSAGE layers, then three layers of graph convolutions are performed before the final READOUT layer. Since the DIFFPOOL layer will reduce the number of nodes by 90%, which makes it impossible for the node classification task, we extract the features matrix from the GRAPHSAGE layer before the DIFFPOOL layer and utilize each row in the matrix as the node representation, which is shown in Figure 2.

For this reason, in the multi-task DIFFPOOL (MT-DIFFPOOL), only parameters in the first two GRAPHSAGE layers are shared. The backpropagation of the graph classification loss starts from the last layer of the network, and the vanishing gradient problem leads to slower learning in the first few layers, thus their parameters may be dominated by the node classification task. These GRAPHSAGE layers before the pooling layer aim to learn efficient node representations, therefore the node classification task could facilitate capturing enhanced node features.

### 3.3. Complexity Analysis

Although applying multi-task framework requires additional computation of the node classification loss, we observed that the MT-GIN and the MT-DIFFPOOL do not incur substantial additional running time compared with GIN and DIFFPOOL in practice. Specifically, for the DIFFPOOL model, the computing cost is concentrated on GRAPHSAGE layers and the computation of an assignment matrix in DIFFPOOL layers, whereas the node classification loss is calculated in the first GRAPHSAGE layer, and it introduces only a few additional computation. Suppose *K* is the number of layers. *n* is the total number of nodes. *m* is the total number of edges. *r* is the number of neighbors being sampled for each node, and *d* is the dimensions of the node hidden features remain constant. The time complexity of a GRAPHSAGE layer is *O*(*r*^*K*^ *nd*^2^), and that of the DIFFPOOL algorithm could be denoted as *O*(*n*^2^). Similarly, the time complexity of GIN is *O*(*m*), and our MTRL framework has the same time complexity as them respectively.

## 4. Experiments

In this section, two state-of-the-art models employed with the proposed multi-task learning architecture are compared with the single-task learning ones. We evaluate the algorithms on an unsupervised learning task: visualization, and two supervised learning tasks: graph classification and node classification. Before we analyze the effect of the presented framework, we first introduce the datasets and model configurations.

### 4.1. Datasets

We use five bioinformatics graph classification benchmarks. For the ENZYMES dataset, the nodes have feature vectors, while for the other datasets, we set the adjacency matrix as input features since that have no features. The statistics of datasets are summarized in Table 2, and details of datasets are as following:

**Table 2:** Statistics of datasets used in our experiments.

**Datasets**	**MUTAG**	**PTC**	**ENZYMES**	**PROTEINS**	**NCI1**
Num. of Graphs	188	344	600	1113	4110
Avg. Number of Nodes	14.29	25.56	32.63	39.06	29.87
Avg. Number of Edges	14.69	25.96	62.14	72.82	32.30
Node Attr. (Dim.)	–	–	+(18)	+(1)	–
Num. of Graph Classes	2	2	6	2	2
Num. of Node Classes	7	19	3	3	37

MUTAG (Debnath et al., 1991) is a dataset of 188 mutagenic aromatic and heteroaromatic nitro compounds, and the classification is based on whether or not they have a mutagenic effect on the Gram-negative bacterium Salmonella typhimurium.

PTC (Predictive ToxicologyChallenge) dataset (Toivonen et al., 2003) contains 344 chemical compounds tested for carcinogenicity in mice and rats. The classification task is to predict the carcinogenicity of the chemical compounds.

ENZYMES (Borgwardt et al., 2005) is a dataset of protein tertiary structures consisting of 600 enzymes from the BRENDA enzyme database (Schomburg et al., 2004). In this case, the task is to correctly assign each enzyme to one of the six EC top-level classes.

PROTEINS (Dobson and Doig, 2003) is similar to ENZYMES, where nodes are secondary structure elements. If two nodes are neighbors in the amino acid sequence or 3D space, there will be an edge between them. Each node has a discrete type attribute (helix, sheet or turn). Different from ENZYMES, it comes with the task of classifying into enzymes and non-enzymes.

NCI1 (Wale et al., 2008) represents a balanced subset of chemical compounds screened for activity against non-small cell lung cancer. This dataset contains more than 4,000 chemical compounds, each of which has a class label between positive and negative. Each chemical compound is represented as an undirected graph where nodes, edges and node labels correspond to atoms, chemical bonds, and atom types respectively.

### 4.2. Model Configurations

In our experiments, we evaluate the MTRL framework on GIN and DIFFPOOL model. Following (Yanardag and Vishwanathan, 2015; Niepert et al., 2016), we report the average of validation accuracy across the 10 folds within the cross-validation. For DIFFPOOL and MT-DIFFPOOL, the mean variant is used in GRAPHSAGE layers, and the *l*_2_ normalization is added to the node embeddings at each layer to make the training more stable. For GIN and MT-GIN, ϵ in Equation (1) is fixed to 0, since this variant is proved to have strong empirical performance (Xu et al., 2019). Batch normalization (Ioffe and Szegedy, 2015) is applied for each layer in the two models. All models are trained for 350 epochs and 10 iterations for each epoch. We use the Adam optimizer (Kingma and Ba, 2015) with the initial learning rate 0.01 and decay it by 0.5 every 50 epochs. Besides, the hyperparameter we tune is the weight of the node classification task α ∈ {0, 0.5, 0.75, 1.25, 1.5, 2}.

## 5. Results

### 5.1. Visualization

Visualizations are indispensable for analyzing high-dimensional data, which is able to intuitively reveal the intrinsic structure of data. Graphs and nodes of a smaller dataset, MUTAG, are represented as representation vectors with different models, and these vectors are further mapped into a two-dimensional space using t-SNE (Maaten and Hinton, 2008).

**Figure 3:**
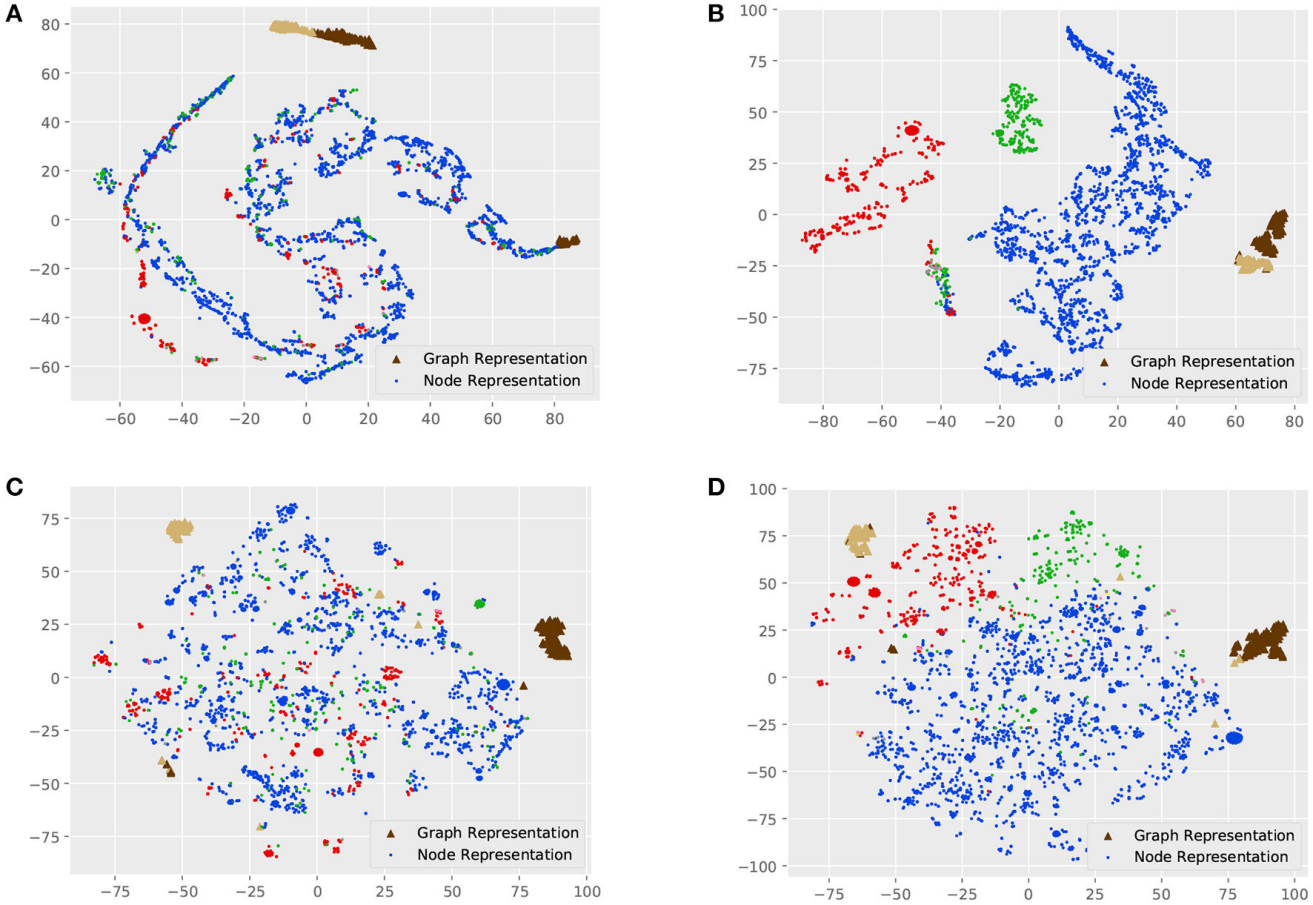
Visualization of the MUTAG dataset. Each point represents a node in the dataset, and triangles of different colors represent graphs of different classes. **(A)** GIN, **(B)** MT-GIN, **(C)** DIFFPOOL, **(D)** MT-DIFFPOOL.

Figure 3 shows the visualization of graph and node representations. For MT-GIN and MT-DIFFPOOL, the hyperparameter α is fixed to 1. There are obvious differences between GIN and DIFFPOOL, as GIN could distinguish the graph representations from the node representations, while graph representations of different classes learned by DIFFPOOL are further away. All models are able to learn distinguishable graph representations, whereas GIN has a part of outliers on the right side and the same thing happens with DIFFPOOL in the lower left corner. In contrast, MT-GIN and MT-DIFFPOOL achieve more compact clusters. These models differ greatly in the performance of node representation learning. The node visualization results of GIN and DIFFPOOL are not meaningful, in which nodes with different tags are clustered together. Models with the MTRL framework achieve superior performance on node visualization, and both MT-GIN and MT-DIFFPOOL form clear boundaries among three main classes of nodes. Intuitively, this experiment demonstrates that the MTRL framework could help learn more meaningful and robust representations.

### 5.2. Training Set Performance

**Figure 4:**
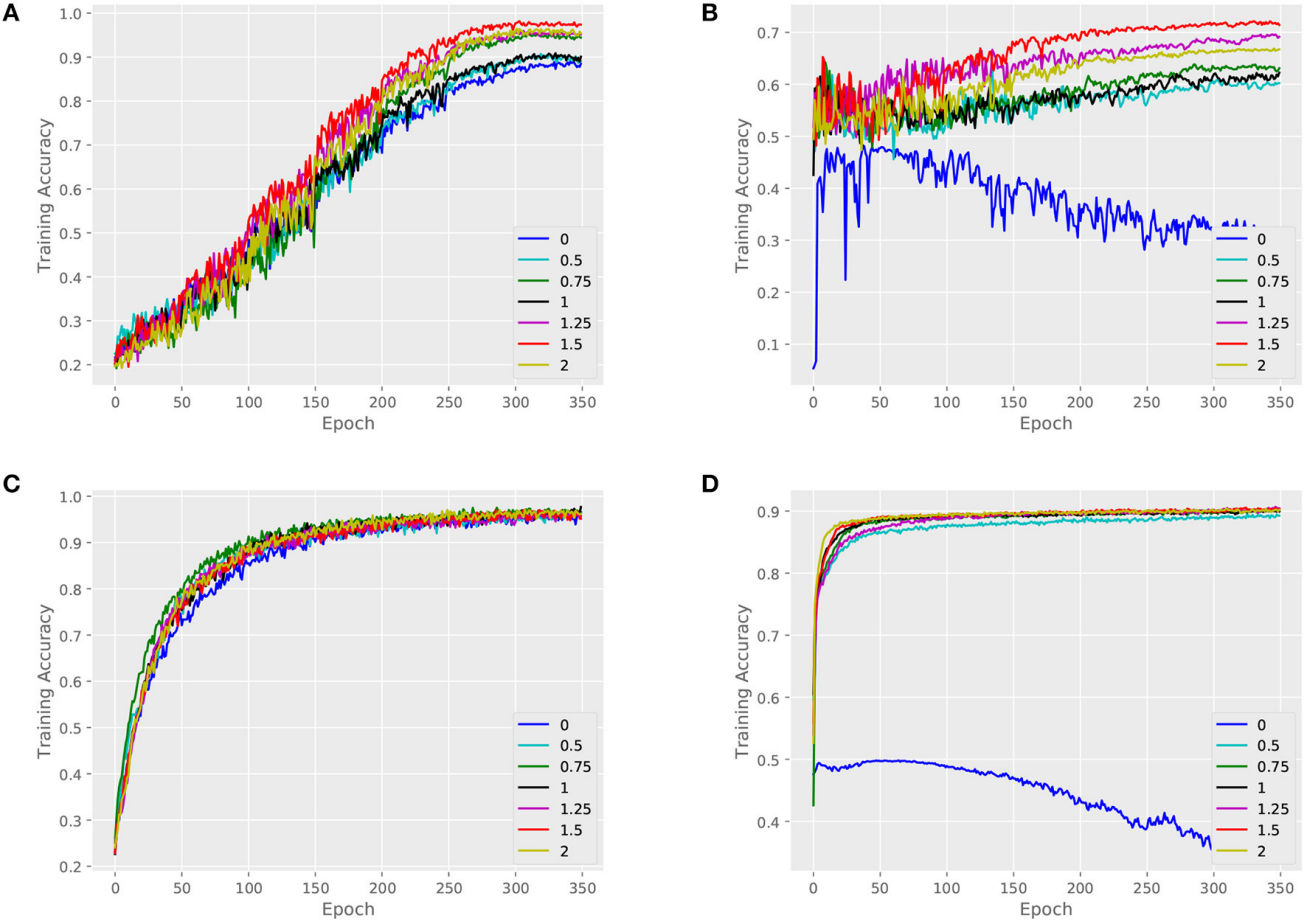
Training set performance of different models on the ENZYMES dataset. **(A)** Training loss for graphs of MT-GIN. **(B)** Training loss for nodes of MT-GIN. **(C)** Training loss for graphs of MT-DIFFPOOL. **(D)** Training loss for nodes of MT-DIFFPOOL.

**Figure 5:**
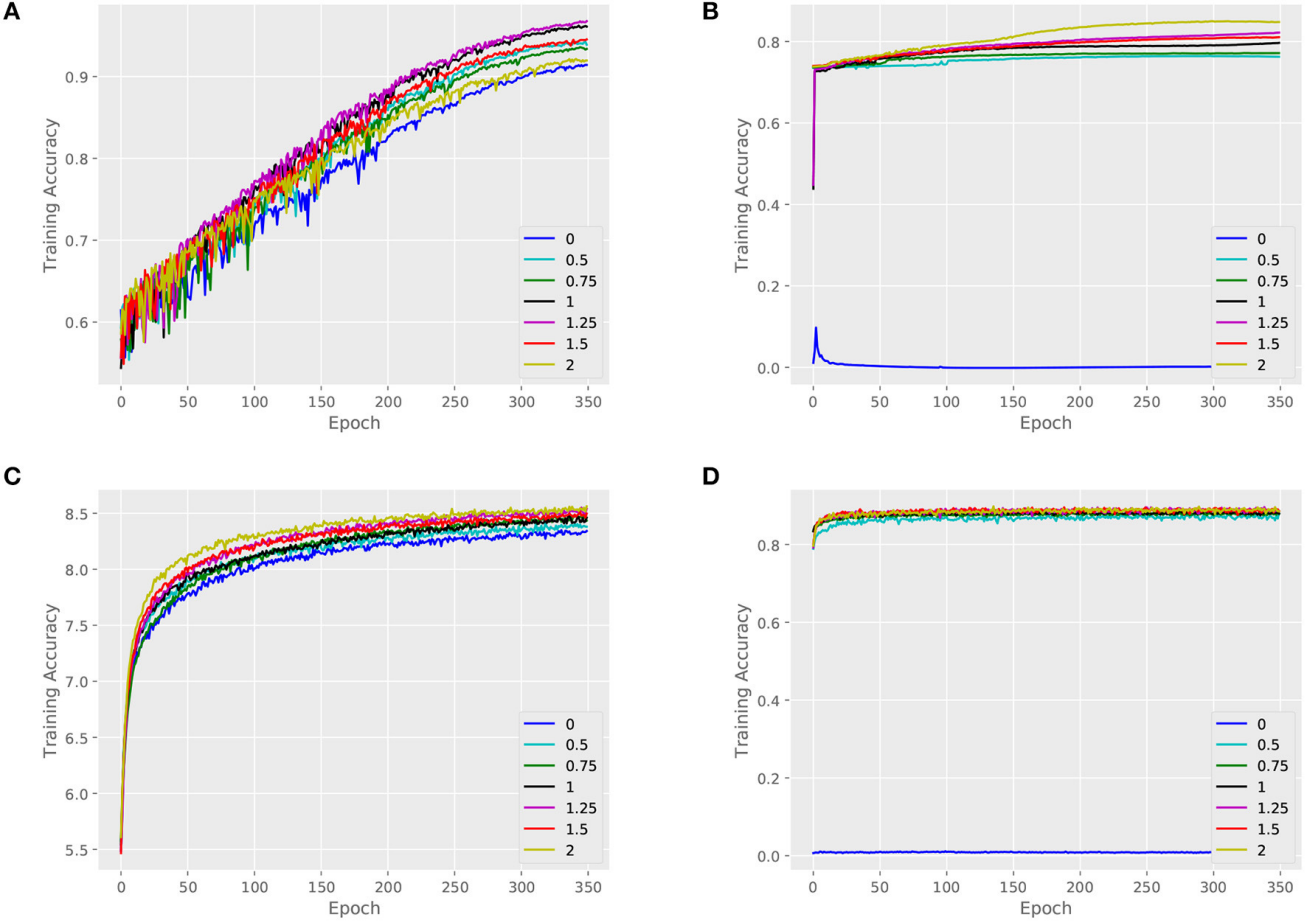
Training set performance of different models on the NCI1 dataset. **(A)** Training loss for graphs of MT-GIN. **(B)** Training loss for nodes of MT-GIN. **(C)** Training loss for graphs of MT-DIFFPOOL. **(D)** Training loss for nodes of MT-DIFFPOOL.

We validate the performance of our architecture and baselines by comparing their training accuracies, and we measure the effect of the key parameter α. An attributed dataset – ENZYMES and a large dataset – NCI1 are taken as examples. Figures 4, 5 show training curves of MT-GIN and MT-DIFFPOOL with different α, noted that the multi-task architecture is equal to a single-task graph classification model when α is 0. In our experiments, the multi-task learning model has a relatively rapid convergence rate, and they brings gain in fitting training compared to fixing α to 0 as in MT-GIN (MIN-0) and MT-DIFFPOOL (DIFFPOOL-0). It should be noted that the node classification accuracy of the MIN-0 and DIFFPOOL-0 tends to decline as iteration increases on ENZYMES, as latent representations of nodes are learned to fit the graph classification task. In particular, the training accuracy aligns with the models' representation power, and the multi-task learning models with different α tend to have higher training accuracies than the single-task learning ones. Moreover, the weight of node classification loss is not always positively correlated with the training accuracy for graphs or nodes, thus the hyperparameter α is important and should be well tuned.

#### 5.2.1. Test Set Performance

Next, we compare test accuracies. We fix the training ratio to 90% and display the average accuracy of graph classification and node classification, as shown in Tables 3, 4. The MTRL architecture consistently outperforms the original GNN models, and it is able to efficiently capture graph structure and node features. By means of node classification task that accurately extracts node attributes, the MTRL architecture can achieve better performance in graph classification.

**Table 3:** Graph classification accuracy (%) of the MTRL architecture as well as the state-of-the-art baselines.

**Datasets**	**MUTAG**	**PTC**	**ENZYMES**	**PROTEINS**	**NCI1**
GIN	89.55	69.71	65.67	73.29	77.12
MT-GIN	**91.63**	**72.65**	**69.55**	**75.48**	**82.59**
DIFFPOOL	87.21	65.04	62.68	72.08	68.91
MT-DIFFPOOL	**87.36**	**70.52**	**64.90**	**76.18**	**71.26**

*The best results are shown in bold*.

**Table 4:** Node classification accuracy (%) of the MTRL architecture as well as the state-of-the-art baselines.

**Datasets**	**MUTAG**	**PTC**	**ENZYMES**	**PROTEINS**	**NCI1**
GIN	28.21	18.60	27.33	26.49	2.08
MT-GIN	**94.35**	**91.02**	**71.23**	**61.85**	**80.48**
DIFFPOOL	19.76	3.11	31.87	29.27	1.22
MT-DIFFPOOL	**97.20**	**88.33**	**82.71**	**73.02**	**83.99**

*The best results are shown in bold*.

For graph classification, both MT-GIN and MT-DIFFPOOL outperform the original models on all datasets. The MUTAG dataset is relatively small with simple structure thus the improvement is not obvious. Specifically, even if node adjacency vectors are provided as input features, it still reaches higher accuracy on PTC and NCI1 dataset. The experimental results demonstrate that models' generalization performance is improved as the potential information contained in multiple tasks is leveraged.

For node classification, it is observed that the MTRL architecture shows significant improvement on five protein datasets, since the results of single-task GNN models are hardly better than random guesses, and their accuracy is relative to the number of nodes in each class. The training accuracy of node classification is very close to the test accuracy on ENZYMES and NCI1, which means the learning of graph-level structure is able to prevent the overfitting of fine-grained node-level features from a macroscopical view.

## 6. Conclusion

In this paper, we develop a novel multi-task representation learning architecture coupled with the task of supervised node classification for enhanced graph classification. Along the way, we extend the architecture to two state-of-the-art GNN models, thus the model could perform node classification during the process of graph classification. We conduct extensive experiments on multiple benchmark datasets, and the experimental results demonstrate that the proposed architecture performs significantly better than various superior GNN methods for graph classification as well as node classification.

Moreover, we will explore the following directions in the future:

(1) The MTRL architecture could simultaneously optimize graph classification and node classification task, and we will make it scalable for other graph applications such as unsupervised link prediction or community detection.

(2) We have analyzed the effect of the weight parameter α, and we plan to explore a self-adaptive parameter or structure that could balance losses of each task. Moreover, it would also be interesting to investigate soft parameter sharing or regularization-based sharing.

## Data Availability Statement

The datasets for this study can be found in the TU Dortmund at https://ls11-www.cs.tu-dortmund.de/staff/morris/graphkerneldatasets.

## Author Contributions

YX and MG conceptualized the problem and the technical framework. MG and YG developed the algorithms and supervised the experiments and exported the data. YX, AQ, and XF implemented the multi-task representation learning architecture simulation. MG managed the project. All authors wrote the manuscript, discussed the experimental results and commented on the manuscript.

### Conflict of Interest

The authors declare that the research was conducted in the absence of any commercial or financial relationships that could be construed as a potential conflict of interest.

## References

[B1] Bengio Y. Courville A. Vincent P. (2013). Representation learning: a review and new perspectives. IEEE Trans. Pattern Anal. Mach. Intell. 35, 1798–1828. 10.1109/TPAMI.2013.50 23787338

[B2] Borgwardt K. M. Ong C. S. Schönauer S. Vishwanathan S. V. Smola A. J. Kriegel H. P. (2005). Protein function prediction via graph kernels. Bioinformatics 21, i47–i56. 10.1093/bioinformatics/bti1007 15961493

[B3] Butepage J. Black M. J. Kragic D. Kjellstrom H. (2017). Deep representation learning for human motion prediction and classification, in Proceedings of the IEEE Conference on Computer Vision and Pattern Recognition (Honolulu, HI), 6158–6166. 10.1109/CVPR.2017.173

[B4] Cao S. Lu W. Xu Q. (2016). Deep neural networks for learning graph representations, in Proceedings of the 30th AAAI Conference on Artificial Intelligence (Phoenix), 1145–1152.

[B5] Chen J. Qiu X. Liu P. Huang X. (2018). Meta multi-task learning for sequence modeling, in Proceedings of the 32nd AAAI Conference on Artificial Intelligence (New Orleans, LA), 5070–5077.

[B6] Choi Y. Choi M. Kim M. Ha J. Kim S. Choo J. (2018). Stargan: unified generative adversarial networks for multi-domain image-to-image translation, in Proceedings of the IEEE Conference on Computer Vision and Pattern Recognition (Salt Lake City, UT), 8789–8797.

[B7] Dai H. Dai B. Song L. (2016). Discriminative embeddings of latent variable models for structured data, in Proceedings of the 33rd International Conference on Machine Learning (New York, NY), 2702–2711.

[B8] Debnath A. K. Lopez de Compadre R. L. Debnath G. Shusterman A. J. Hansch C. (1991). Structure-activity relationship of mutagenic aromatic and heteroaromatic nitro compounds. correlation with molecular orbital energies and hydrophobicity. J. Med. Chem. 34, 786–797. 1995902 10.1021/jm00106a046

[B9] Defferrard M. Bresson X. Vandergheynst P. (2016). Convolutional neural networks on graphs with fast localized spectral filtering, in Advances in Neural Information Processing Systems (Barcelona) 3844–3852.

[B10] Dobson P. D. Doig A. J. (2003). Distinguishing enzyme structures from non-enzymes without alignments. J. Mol. Biol. 330, 771–783. 10.1016/S0022-2836(03)00628-4 12850146

[B11] Du X. Wang J. J. (2015). Support image set machine: jointly learning representation and classifier for image set classification. Knowl Based Syst. 78, 51–58. 10.1016/j.knosys.2015.01.016

[B12] Duvenaud D. K. Maclaurin D. Iparraguirre J. Bombarell R. Hirzel T. Aspuru-Guzik A. (2015). Convolutional networks on graphs for learning molecular fingerprints, in Advances in Neural Information Processing Systems (Montreal, QC), 2224–2232.

[B13] Feng L. Zhou L. Zhong J. Gupta A. Ong Y.-S. Tan K.-C.. (2018). Evolutionary multitasking via explicit autoencoding. IEEE Trans. Cybernet. 49, 3457–3470. 10.1109/TCYB.2018.2845361 29994415

[B14] Gilmer J. Schoenholz S. S. Riley P. F. Vinyals O. Dahl G. E. (2017). Neural message passing for quantum chemistry, in Proceedings of the 34th International Conference on Machine Learning (Sydney, NSW), 1263–1272.

[B15] Hamilton W. Ying Z. Leskovec J. (2017a). Inductive representation learning on large graphs, in Advances in Neural Information Processing Systems (Long Beach, CA), 1024–1034.

[B16] Hamilton W. L. Ying R. Leskovec J. (2017b). Representation learning on graphs: methods and applications. IEEE Data Eng. Bull. 40, 52–74.

[B17] Huang S. Zhong J. Yu W. (2019). Surrogate-assisted evolutionary framework with adaptive knowledge transfer for multi-task optimization. IEEE Trans. Emerg. Top. Comput. 10.1109/TETC.2019.2945775 [Epub ahead of print].

[B18] Ioffe S. Szegedy C. (2015). Batch normalization: accelerating deep network training by reducing internal covariate shift, in Proceedings of the 32nd International Conference on Machine Learning (Lille), 448–456.

[B19] Janner M. Narasimhan K. Barzilay R. (2018). Representation learning for grounded spatial reasoning. Trans. Assoc. for Comput. Linguist. 6, 49–61. 10.1162/tacl-a-00004

[B20] Kendall A. Gal Y. Cipolla R. (2018). Multi-task learning using uncertainty to weigh losses for scene geometry and semantics, in Proceedings of the IEEE Conference on Computer Vision and Pattern Recognition (Salt Lake City, UT), 7482–7491.

[B21] Kingma D. P. Ba J. (2015). Adam: a method for stochastic optimization, in Proceedings of the 3rd International Conference on Learning Representations.

[B22] Kipf T. N. Welling M. (2016). Semi-supervised classification with graph convolutional networks, in Proceedings of the 4th International Conference on Learning Representations.

[B23] Lei T. Jin W. Barzilay R. Jaakkola T. (2017). Deriving neural architectures from sequence and graph kernels, in Proceedings of the 34th International Conference on Machine Learning (Sydney, NSW), 2024–2033.

[B24] Li Y. Tarlow D. Brockschmidt M. Zemel R. (2016). Gated graph sequence neural networks, in Proceedings of the 4th International Conference on Learning Representations (New York, NY).

[B25] Liu S. Johns E. Davison A. J. (2019). End-to-end multi-task learning with attention, in Proceedings of the IEEE Conference on Computer Vision and Pattern Recognition (San Juan), 1871–1880.

[B26] Maaten L. V. D. Hinton G. (2008). Visualizing data using t-sne. J. Mach. Learn. Res. 9, 2579–2605.

[B27] Monti F. Bronstein M. Bresson X. (2017). Geometric matrix completion with recurrent multi-graph neural networks, in Advances in Neural Information Processing Systems (Long Beach, CA), 3697–3707.

[B28] Niepert M. Ahmed M. Kutzkov K. (2016). Learning convolutional neural networks for graphs, in Proceedings of the 33rd International Conference on Machine Learning (New York, NY), 2014–2023.

[B29] Petar V. Guillem C. Arantxa C. Adriana R. Pietro L. Yoshua B. (2018). Graph attention networks, in Proceedings of the 6th International Conference on Learning Representations (Vancouver, BC).

[B30] Rossi R. A. McDowell L. K. Aha D. W. Neville J. (2012). Transforming graph data for statistical relational learning. J. Artif. Intell. Res. 45, 363–441. 10.1613/jair.3659

[B31] Sanh V. Wolf T. Ruder S. (2019). A hierarchical multi-task approach for learning embeddings from semantic tasks, in Proceedings of the 33rd AAAI Conference on Artificial Intelligence (Honolulu, HI).

[B32] Scarselli F. Gori M. Tsoi A. C. Hagenbuchner M. Monfardini G. (2008). The graph neural network model. IEEE Trans. Neural Netw. 20, 61–80. 10.1109/TNN.2008.2005605 19068426

[B33] Schlichtkrull M. Kipf T. N. Bloem P. Van Den Berg R. Titov I. Welling M. (2018). Modeling relational data with graph convolutional networks, in Proceedings of the 15th European Semantic Web Conference (Heraklion), 593–607.

[B34] Schomburg I. Chang A. Ebeling C. Gremse M. Heldt C. Huhn G.. (2004). Brenda, the enzyme database: updates and major new developments. Nucleic Acids Res. 32, D431–D433. 10.1093/nar/gkh081 14681450 PMC308815

[B35] Schulz C. Eger S. Daxenberger J. Kahse T. Gurevych I. (2018). Multi-task learning for argumentation mining in low-resource settings, in Proceedings of the 2018 Conference of the North American Chapter of the Association for Computational Linguistics: Human Language Technologies (New Orleans), 35–41.

[B36] Toivonen H. Srinivasan A. King R. D. Kramer S. Helma C. (2003). Statistical evaluation of the predictive toxicology challenge 2000–2001. Bioinformatics 19, 1183–1193. 10.1093/bioinformatics/btg130 12835260

[B37] Tran P. V. (2018). Learning to make predictions on graphs with autoencoders, in Proceedings of the 5th IEEE International Conference on Data Science and Advanced Analytics (Turin), 237–245.

[B38] Wale N. Watson I. A. Karypis G. (2008). Comparison of descriptor spaces for chemical compound retrieval and classification. Knowl. Inf. Syst. 14, 347–375. 10.1007/s10115-007-0103-5

[B39] Xu K. Hu W. Leskovec J. Jegelka S. (2019). How powerful are graph neural networks? in Proceedings of the 7th International Conference on Learning Representations.

[B40] Xu K. Li C. Tian Y. Sonobe T. Kawarabayashi K. Jegelka S. (2018). Representation learning on graphs with jumping knowledge networks, in Proceedings of the 35th International Conference on Machine Learning (New Orleans, LA), 5453–5462.

[B41] Yanardag P. Vishwanathan S. (2015). Deep graph kernels, in Proceedings of the 21th ACM SIGKDD International Conference on Knowledge Discovery and Data Mining (New York, NY), 1365–1374.

[B42] Yang M. Simm J. Lam C. C. Zakeri P. van Westen G. J. P. Moreau Y.. (2018). Linking drug target and pathway activation for effective therapy using multi-task learning. Sci. Rep. 8:8322. 10.1038/s41598-018-25947-y 29844324 PMC5974390

[B43] Ying Z. You J. Morris C. Ren X. Hamilton W. Leskovec J. (2018). Hierarchical graph representation learning with differentiable pooling, in Advances in Neural Information Processing Systems (Montreal, QC), 4800–4810.

[B44] Zhang M. Cui Z. Neumann M. Chen Y. (2018). An end-to-end deep learning architecture for graph classification, in Proceedings of the 32nd AAAI Conference on Artificial Intelligence (New Orleans, LA), 4438–4445.

[B45] Zou D. Lerman G. (2019). Graph convolutional neural networks via scattering. Appl. Comput. Harmon. Anal. 10.1016/j.acha.2019.06.003

